# Supplementary figures and images for: Co-transcriptional splicing facilitates transcription of gigantic genes
Source: PLoS Genet. 2024 Jun 13;20(6):e1011241. doi: 10.1371/journal.pgen.1011241 (PMC11207136; doi:10.1371/journal.pgen.1011241)

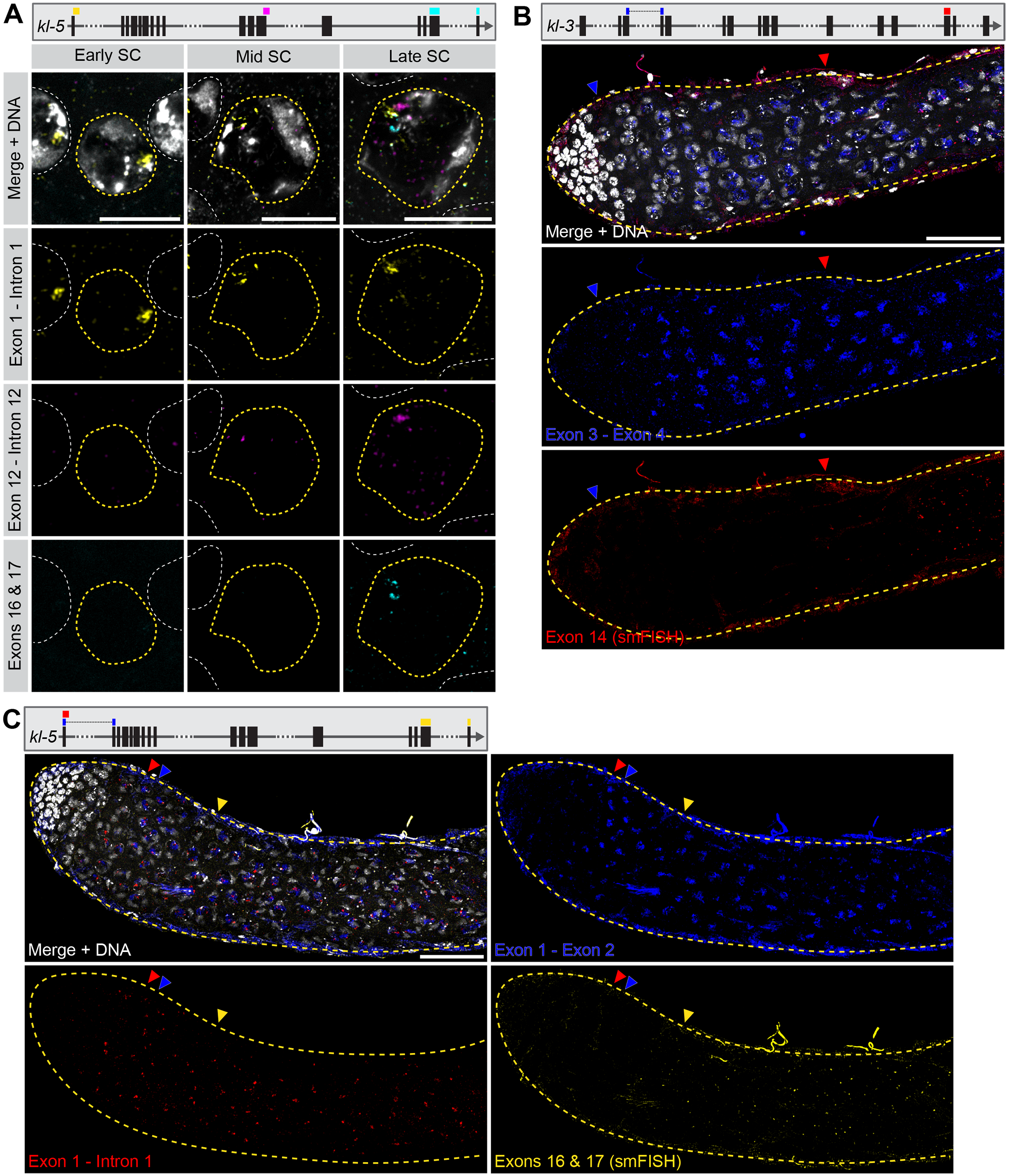

Supplement: S1 Fig — (A) Top: kl-5 gene diagram showing probe target locations. Bottom: HCR RNA FISH in wildtype SCs of increasing maturity [single SC nuclei (yellow dashed line), neighboring SC nuclei (white dashed line). Exon 1 –intron 1 (yellow), exon 12 –intron 12 (magenta), exons 16 & 17 (smFISH, cyan), DNA (white). Bars: 10μm. (B) Top: kl-3 gene diagram showing probe target locations. Bottom: HCR RNA FISH in wildtype testes (yellow dashed outline). Exon 3 –exon 4 (blue), exon 14 (smFISH, red), DNA (white). Colored arrowheads indicate earliest detection of each probe. Bar: 50μm. (c) Top: kl-5 gene diagram showing probe target locations. Bottom: HCR RNA FISH in wildtype testes (yellow dashed outline). Exon 1 –intron 1 (red), exon 1 –exon 2 (blue), exons 16 & 17 (smFISH, yellow), DNA (white). Colored arrowheads indicate earliest detection of each probe. Bar: 50μm. (TIF) [file pgen.1011241.s001.tif]

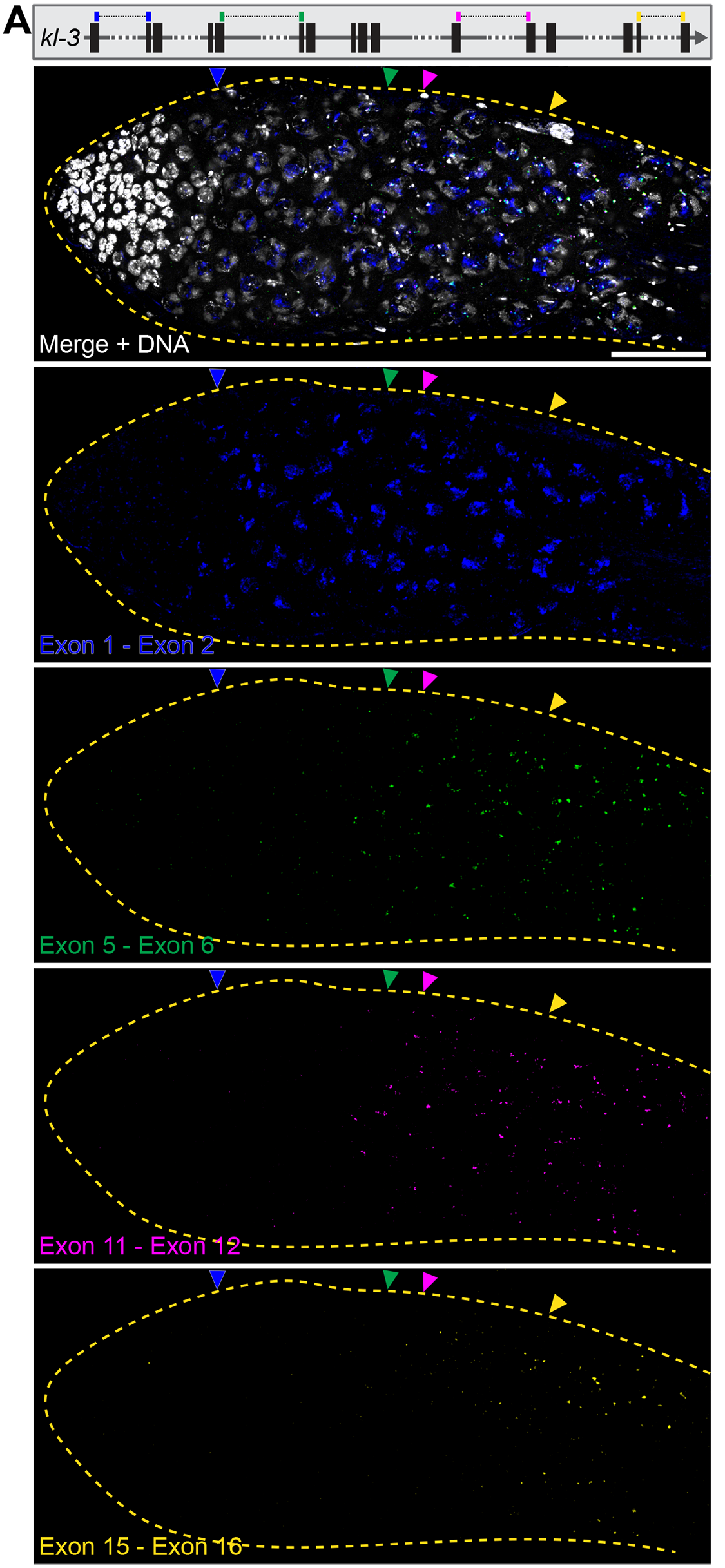

Supplement: S2 Fig — (A) Top: kl-3 gene diagram showing probe target locations. Bottom: HCR RNA FISH in wildtype testes (yellow dashed outline). Exon 1 –exon 2 (blue), exon 5 –exon 6 (green), exon 11 –exon 12 (magenta), exon 15 –exon 16 (yellow), DNA (white). Colored arrowheads indicate earliest detection of each probe. Bar: 50μm. (TIF) [file pgen.1011241.s002.tif]

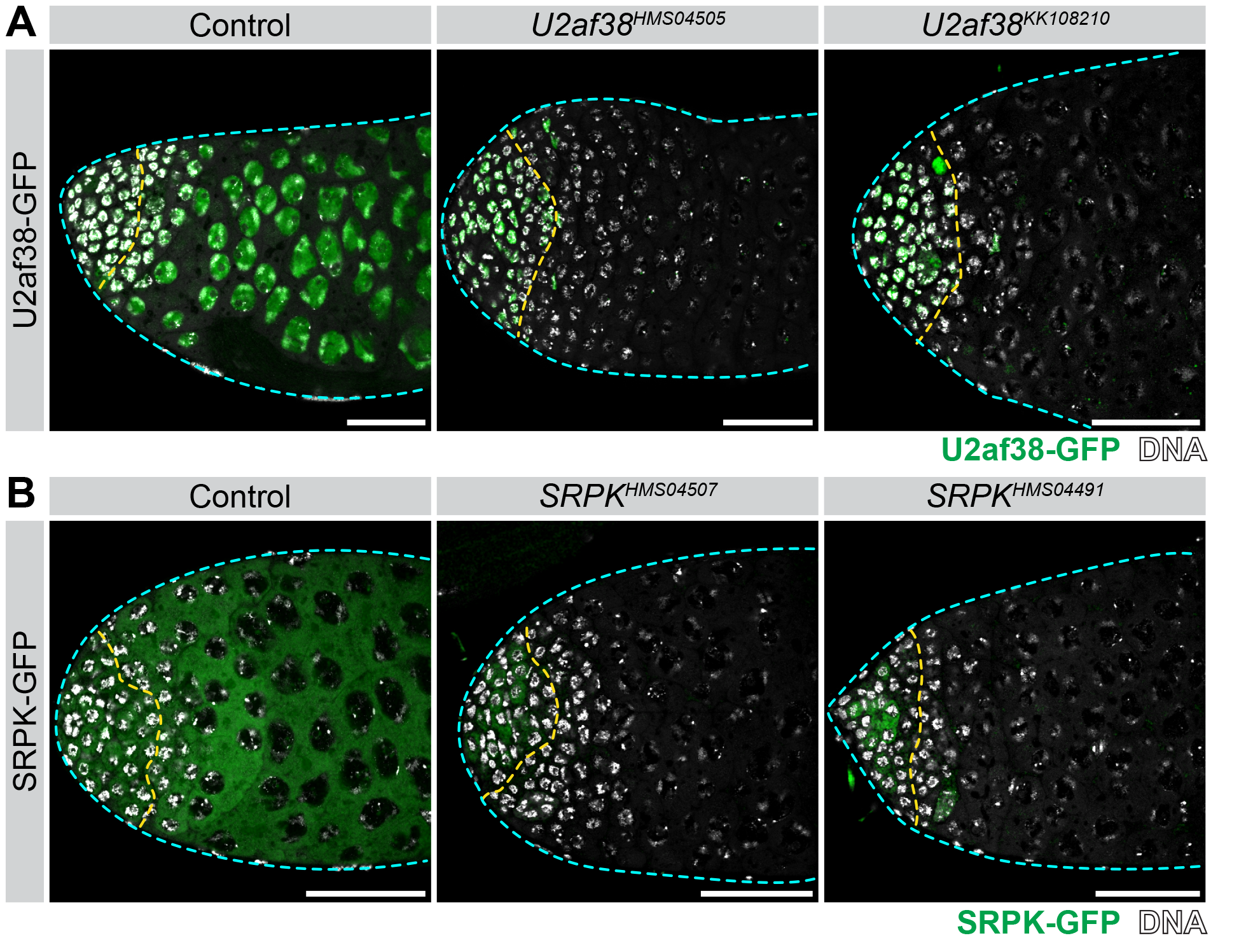

Supplement: S3 Fig — (A) U2af38-GFP expression in the apical tip of the testis (cyan dashed line) in the indicated genotypes. bam driven RNAi expression starts part way through spermatogonial differentiation (yellow dashed line), leaving the germline stem cells/early spermatogonia unaffected. Bars: 50μm. (B) SRPK-GFP expression in the apical tip of the testis (cyan dashed line) in the indicated genotypes. bam driven RNAi expression starts part way through spermatogonial differentiation (yellow dashed line), leaving the germline stem cells/early spermatogonia unaffected. Bars: 50μm. (TIF) [file pgen.1011241.s003.tif]

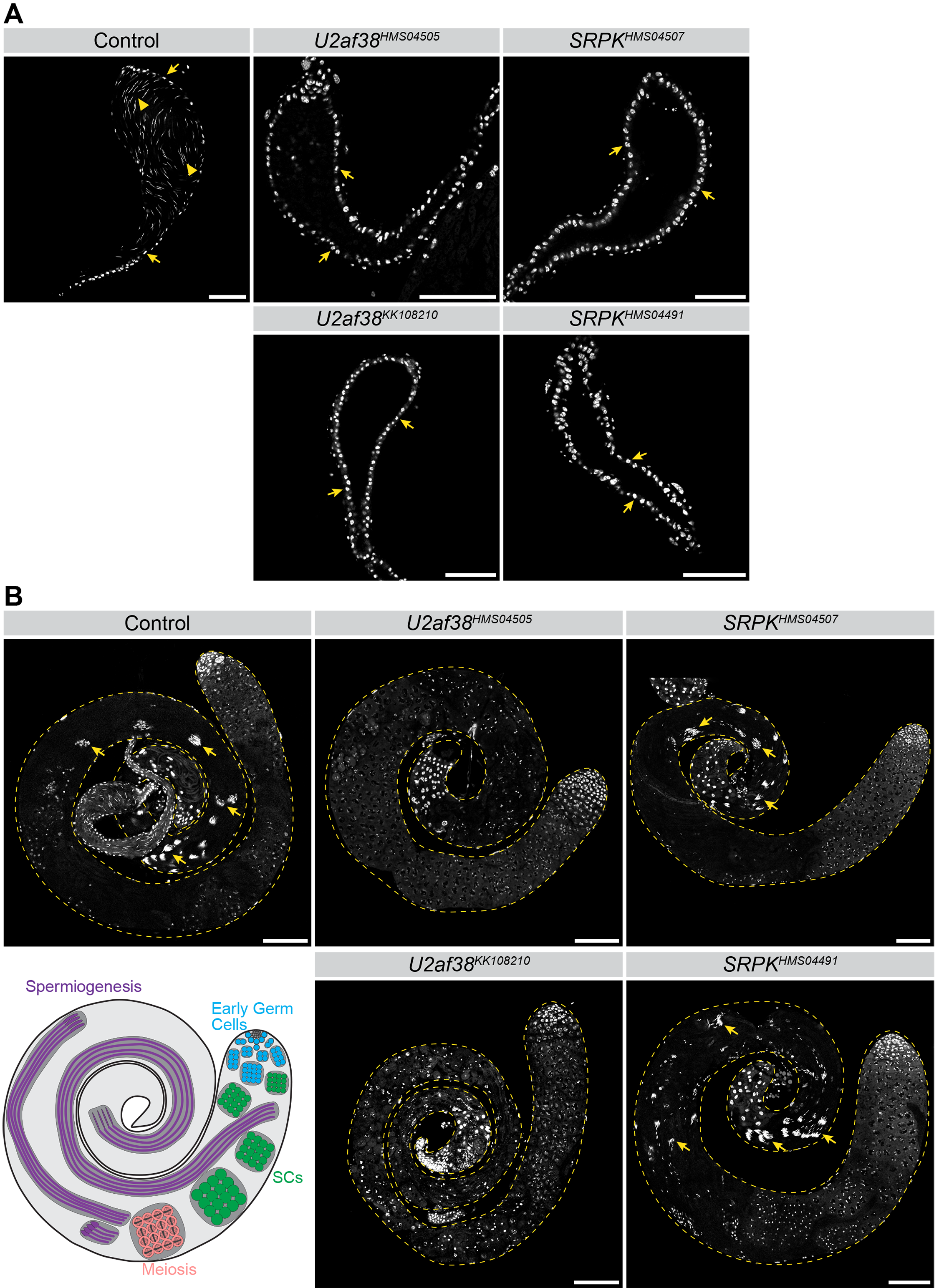

Supplement: S4 Fig — (A) Seminal vesicles in the indicated genotypes. DNA (white). Bars: 50μm. Yellow arrows indicate round epithelial cells while yellow arrowheads indicate needle-shaped sperm nuclei. (B) Whole testes (yellow dashed lines) in the indicated genotypes. DNA (white), maturing sperm nuclei (yellow arrows). Diagram (bottom left) illustrates proper germ cell development. Spermiogenesis is absent in U2af38 RNAi testes while SRPK RNAi testes appear phenotypically normal. (TIF) [file pgen.1011241.s004.tif]

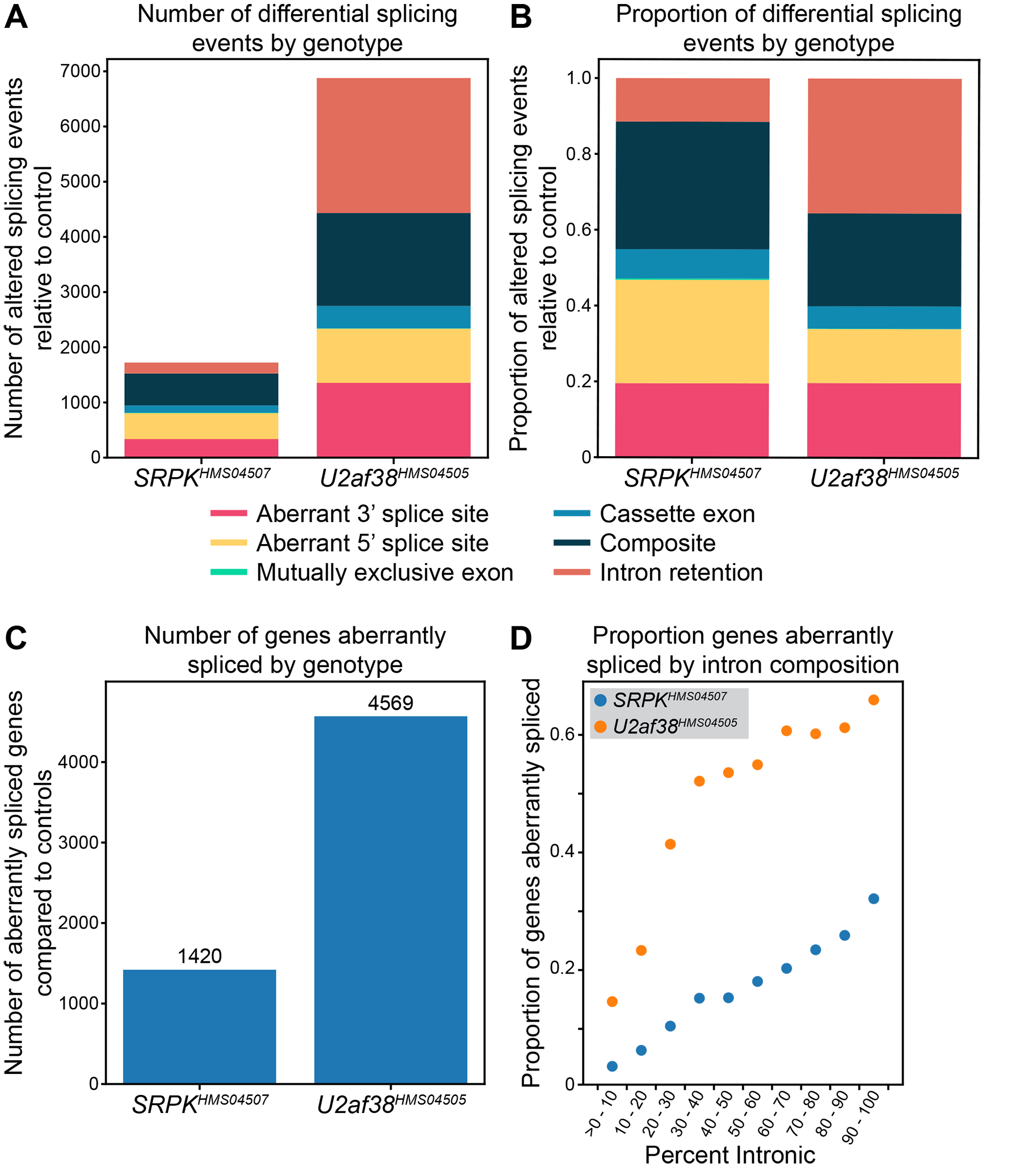

Supplement: S5 Fig — (A and B) Graphical representation of the number (A) and proportion (B) of different types of aberrant splicing events in U2af38 and SRPK RNAi conditions that were detected by JUM (q < 0.05). (C) Total number of genes aberrantly spliced in U2af38 and SRPK RNAi. (D) Graph showing the proportion of genes in each intron proportion bin (percent of the gene span that is intronic) that are aberrantly spliced in U2af38 and SRPK RNAi. (TIF) [file pgen.1011241.s005.tif]

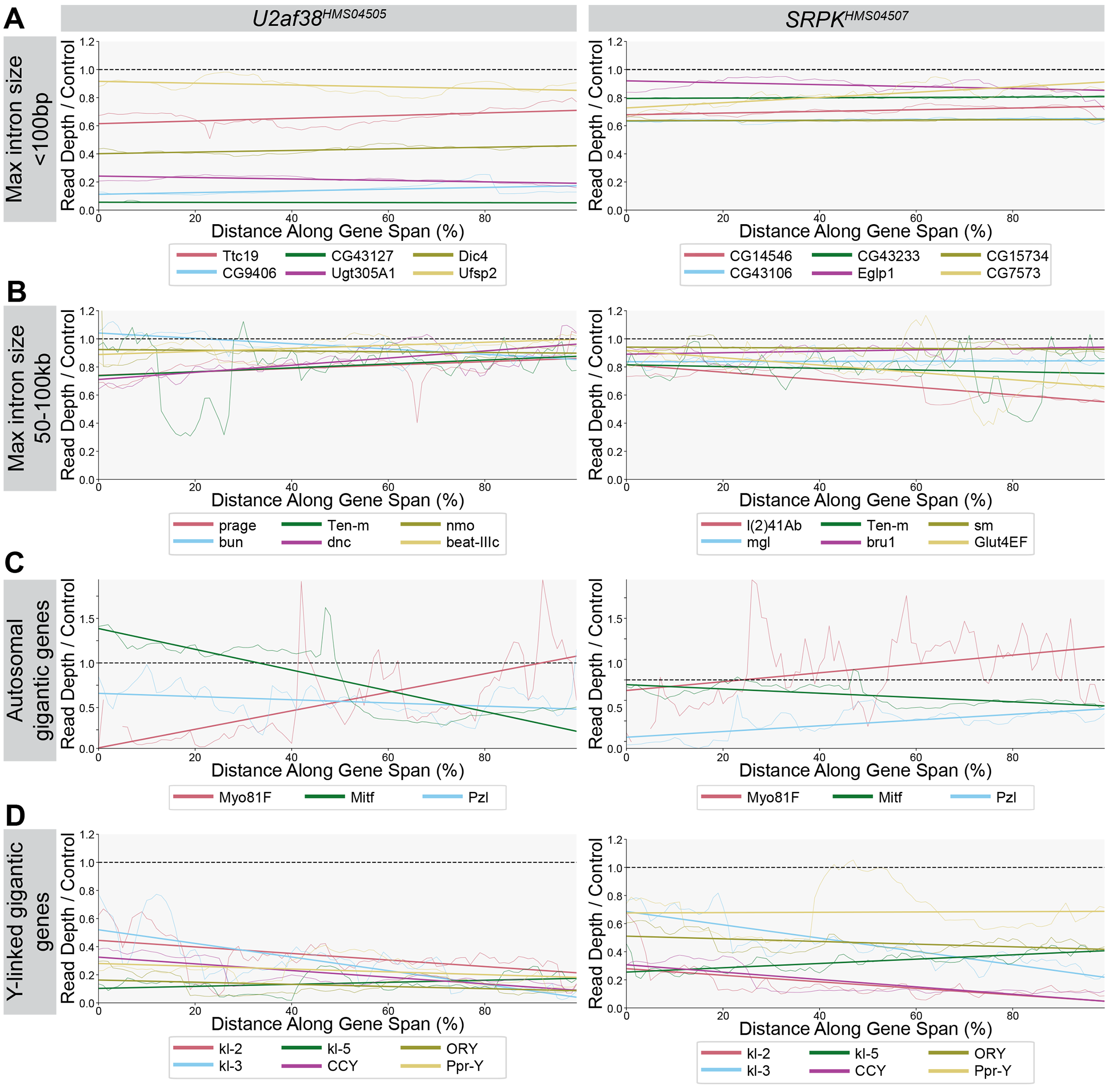

Supplement: S6 Fig — (a–d) Plots showing the coverage along the gene span (exons only, normalized) relative to the control condition for selected aberrantly spliced genes in either U2af38 (left column) or SRPK (right column) RNAi. Linear best fit lines estimate changes in expression over the gene span relative to the control condition. (a) max intron size less than 100bp. (b) max intron size between 50–100kb. (c) Autosomal genes with gigantic introns. (d) The Y-linked gigantic genes. PRY and WDY omitted due to low overall expression and/or errors in the genome annotation (see Methods). (TIF) [file pgen.1011241.s006.tif]
