# Supplementary material for: Co-transcriptional splicing facilitates transcription of gigantic genes
Source: PLoS Genet. 2024 Jun 13;20(6):e1011241. doi: 10.1371/journal.pgen.1011241 (PMC11207136; doi:10.1371/journal.pgen.1011241)
Supplement: S1 Table — (DOCX) [file pgen.1011241.s007.docx]

**smFISH probe sets**

| **Probe Target** | **Fluorophore** | **5’-Sequence-3’** |
| --- | --- | --- |
| *kl-2*, Exons 1 & 2 | Fluorescein | Cgatcagtctcagtactttc, ttcacatattcaacaagccc, tctgttgaattgatcaacca, gtgagaacatcgcaatcgta, acaggaaatccaaggcaacc, cataaatcaataaccggggc, ttatttctttagaggaccga, cgaagttaaccatgtcgtga, caacatcatcttgcacagta, aaattttcaataggcagcca, tgggagcatatataagctct, cccattccataaaatttcga, aacgttgcttcacattgtct, atttatccagggacgtacag, ttgctgttaagattcctaga, aactgtcatgccagacattt, ctcttttgctatttctttga, attctctgtcgtacgatgaa, cgcaatacactccaagttct, aattcgaataagcgtagccc, tggtaagggtcttgtcattt, ctcatccccaacactaatat, accgatatagccagaactcg, tgcgcatttagtccttgaag, ttgaacatccacttgatcca, gggtacttcgtagaactctt, attagttcatcgatctgttt, ccatcagttcatgtgtagat, atttgatgttttccatagct, gttgtgaacatggttgcatt, gtgagacaaaagttgggctt, tcaagtgaattgttcgggga, gtattgttcagagtttaacc, ccagttattaaatcacgtct, ccttttcagtacaaaaccgt, tccaaaccttgaacttcctg, attcctttataagtcaggca, ttttgcatgggtttttgaca, ccaacctattcgtatgttta, aatcattgcattgtcaaggc, ctccataaaggcatcgacat, gtcttccgaaaaccatcatt, gtatactctctgattcgtca, taccaccgaattgaggctta, gcttcaaattctgtaccact, attttcaacgttgtcggcag, ataatgccgtaagagtcacc, atgatctctttggaatccgt |
| *kl-3*, Exon 1 | Quasar® 670 | taacattcctttctggatcc, cgcgaaacgccaaagagttt, gcagcacgctttaacatgtt, ttggtcacttacactaggtc, tatcgtcttctttgttggtc, cctcatttctcgaagtaact, caggcttgaaatccgttgtt, aaacataccgctggtttggg, atacccaacaaatctgcaca, gttactatttcctcaggatc, ttgctttcatccacaatacc, accatttacattctcaacat, gggacctttttcctcaaata, cgttacttatcattatggcc, cggaatcagttggatatcct, tttaagcttttcctggtagc, agagcgttgaatttcagtgt, actagatccgacctcaaaca, gtcgatatacaacagtccac, accgaacggttatcaatcga, aaatattgccacctcatcac, aagcaagagtttcgctcttc, cggctttaaaacgtgatcca, caaattcggtcacagcttct, tgagtttgctctttttctgc, acagttgcttcagatgattt, cctttaaatagttcatgcga, tttgccatctcacaagtaat, gtatttttgaactggcttca, caaccagtcgaactcgtgta, gccattgctcaaaataacgt, gtataccttgaatttgtcga, gtatttgttttccctctact, ctacatcgggtgtatctctt, ccaattgaccaacatttgca, tatatctggccaacatacgc, gtaacaaactccgttatggt, gtggttattaaatgctcggg, agataatgtcaagcaatcct |
| *kl-3*, Exon 14 | Quasar® 570 | gtactttgacatagccatgg, aagatttgcctttaagggca, tgatatttagcctcttgcac, ctgcttcttgtagatcactt, cgttttctttttgttgcagt, tttttggcctcgtctaatac, aaccaccaataagagcggtt, ttcagtccatcggatttttt, cggtcggtctcacttttaaa, ggagaataacatctccgacc, tcttgattaaatggtcccgt, atgttccactcaccaatttg, ttgacagttcatctgttggt, ttttaatccacacttttccc, taatcggaattcccatgcta, taactcctctgcaacatctt, aaggttgtccaagcaaggat, tccaatccacttctttatca, gattgggtagctttgttgta, cgcgcaaatatttcaggtgt, ccacgcattgtcacagtaaa, gaacccgttcatcttctaat, tttcatgtttccagtcacag, ttcctttcgtagtggacaac, cctcaatcactgtaacgtcg, tccttaacttcaatggcagt, cagcgtttatttttgcttct, acactacctcttgtagcaac, gcatcaaagcgctcaaggaa, tcctctagatttgtagcgat, ttaactcaagctggcgatca, cgcaccgccttttataaata, caccgaaacggaacaggagg, tataccacgtaaaccaagcc, ccatacaccacgaacgaaca, agtttagtactactggctcg, aatcattggcataagttccc, agctcattttttttggcgag, cttggcccatagaaatagga, cgtcttctaggcaggataat, tcctaagtgacagttttgca, actgtaagctctaccatgta, agcaggtggttcattagtat, tttttagtccagcacgtatt, tggagattgcgaatagtcca, tgcataccagtcggatgaat, tcctgctccttaaaactgta, cgtttgccatgtcatcaata |
| *kl-5*, Exons 1-6 | Quasar® 570 | cttcttttccttttcgtcag, aaaaactccggacggttgtc, ttgtcttggttaggtagttc, ccacttatccagcttaagac, cctaaactcgttggttgtta, atacgtttcttgttgggatt, ccaccggaattgattgtgaa, ctggaaagctgtaggatgga, agcaactttataccgtggtc, gaagtggtgttaagtaccga, atttgctaatgggttcggta, atcccacttgatttactgtg, tctgtcttcatatcgtttgc, tccatttcgcatttcttgag, aacgagacctttcatctggg, catgacttcgtccatgcaaa, gcttcataagagggttaacc, caagccactttacaaccata, tttacgaggtcttcaacgga, tgcctctggtagtggaaaat, caagttctcaaggttttcca, agtctttatacgcctatctc, tctgcgataagaatgctcga, acttagttatgtctctagcc, gtccatatcgtttgtttcaa, gtcgtatatctgatcggttc, cactaatccaattgtcagca, tgaaaataccgcgagtgacc, gttctgttgacacagtcgat, ggaatatggagtctggttct, ttataggcttcgtcgacatc, tgtaatactctaggtgctgc, cagtcctgcgagttaaatgt, atcgtccgaatatttcatcc, tcttttagctcttccaatcg, ccccataacaattttttcca, aagggcgaaagttatctgcc, tgctgttgtactcttcaaga, aatatttgtccactcacggt, acagagaattttctgcgtcc, tggccttgaagcttaaacga, tgaaacgataccatgcgctc, atgtgtgtcttcaagacctt, gcgaagaagtaaggagcctg, gggttcgacatgttttttga, atcatccacaatgacatgca, taacgcacatgatctcttcc, cgagcgccgtaaaaacgttt |
| *kl-5*, Exons 16-17 | Fluorescein | ttgtggtccgaatttcctac, taaacgggtagctacggttc, tgatattgtcaaatcgccca, ccaggtaattgtacagcaca, caaggtactctggtattggc, ccatacataatctctccgaa, ccagtcgtctgtaatgtgac, gataagttcggcataagcgg, agctctggctgcataaattc, aaaccctggcaatactctag, atttaagtattcctggagcc, atgtaattgtggtagccagt, gagatggactttcagacgga, gcatttgaatgaaggccgta, cgtagttaggaacccaatct, attcggaagagtcgttcaga, tctcggctgcaactcgaaaa, aaactgtctcaccaccacta, gttttttataatgtcttcct, aatggtgtgggagttttgtc, cgcgacccattagttctaaa, atgtatggacttcggtcttc, cgctcacactcttgaaatgc, gcttcaattcagtcataaga, aggtcaagctcattcagaga, agtcaattctcctttaaggc, ccattaaatcctccataact, gcacttggtccatgtaaaga, aaccaggattgtagccctag, cagccgcaacattaaatcgg, aggcatgcgaaagtcggcaa, atcctgccaaccaaattgat, aggagcgactgaggattaaa, cgtctgttgcatgatagctg, gacacattctatcgagaggc, ttccactttttggtgacatc, ttgatataggcaccctctcg, tgctccctccatgaaaagac, aatggttcccattttcatgt, gttcctttaaaaaggcgtct, agaacaggcatagcaggaaa, cttgggttactgcctttatg, gacatttttaatatcctgct, cggattttgtaaactgggca, caaacgaaggtgggtcctcg, ctctcggcttttcaagttaa, ctagagtccacttacttgct, tgcaagagaagacaaacccc |

**RNA FISH probes for repetitive sequences**

| **Probe Target** | **5’-Sequence-3’** |
| --- | --- |
| (AATAT)n | Alexa488-ATATTATATTATATTATATTATATTATATT  Cy5-ATATTATATTATATTATATTATATTATATT |
| (AAGAC)n | Cy3-AAGACAAGACAAGACAAGACAAGACAAGAC  Cy5-AAGACAAGACAAGACAAGACAAGACAAGAC |

**HCR RNA FISH probes**

| **Gene** | **Junction** | **Target Sequence** | **Amplifier** |
| --- | --- | --- | --- |
| *kl-3* | exon1 - intron1 | TCGAAACTTTTAAACgtaagttactttgtc | B3 |
| *kl-3* | intron1 - exon2 | tttttttgtttctagCGCTGGGCTGAAATC | B2 |
| *kl-3* | exon1 - exon2 | TCGAAACTTTTAAACcgctgggctgaaatc | B4 |
| *kl-3* | exon3 - intron3 | GGAAAAAGTGTGCATGgtaagaaattgtttgg | B1 |
| *kl-3* | intron3 - exon 4 | atttttccaaattagAAAATAAAAGAAGTGG | B2 |
| *kl-3* | exon3 - exon4 | GGAAAAAGTGTGCATGaaaataaaagaagtgg | B3 |
| *kl-3* | exon5 - intron5 | GACAAAATATACTCAGgtatggccagcacct | B2 |
| *kl-3* | exon5 - exon6 | GACAAAATATACTCAGaactaattggcgaac | B1 |
| *kl-3* | exon11 - exon12 | CCTAACATGTACCAGagaataattgagtct | B2 |
| *kl-3* | exon15 - intron15 | GACAGAATGCAGAGGgtaagtatggtgaaa | B1 |
| *kl-3* | exon15 - exon16 | GACAGAATGCAGAGGattataattttagtacgg | B3 |
| *kl-5* | exon1 - intron1 | ACCAACGAGTTTAGGgtgagtctaataccc | B4 |
| *kl-5* | exon1 - exon2 | accaacgagtttaggGATGTTATTATAGAG | B3 |
| *kl-5* | exon12 - intron12 | GCCGGAGCTCTTAGGgtaagatattttaca | B2 |
