# Supplementary material for: Co-transcriptional splicing facilitates transcription of gigantic genes
Source: PLoS Genet. 2024 Jun 13;20(6):e1011241. doi: 10.1371/journal.pgen.1011241 (PMC11207136; doi:10.1371/journal.pgen.1011241)
Supplement: Attachment — Submitted filename: Splicing-PLOSG-response.docx [file pgen.1011241.s009.docx]

Reviewer #1: Fingerhut and colleagues report that when splicing is inhibited, very long genes fail to be completely transcribed. Authors analyzed the Drosophila Y-linked gigantic genes that are expressed during spermatocyte development. Using RNA FISH to image spliced and unspliced transcripts, authors show that exons 1 and 2 are ligated before exon 14 is transcribed, which occurs days later. This observation is consistent with the prevailing model that splicing occurs co-transcriptionally. Next, authors used RNAi to downregulate critical splicing factors (U2af38 and SRPK) and showed that both are required for proper sperm formation. Moreover, splicing was inhibited, as expected. Most interestingly, transcripts ceased to be detected beyond the very long intronic regions, suggesting an impairment of transcription elongation. However, authors do not elucidate the mechanism underlying this phenomenon. Instead, they propose a somewhat nebulous model suggesting that transcription is hindered by the presence of tangled, unspliced long pre-mRNAs.
Notably, it has been previously shown that PlaB, a small molecule splicing inhibitor, induces premature transcription termination, predominantly in long genes (doi: 10.1016/j.molcel.2021.02.034; see Fig. 4). Treatment with PlaB was also shown to cause massive abortion of transcription of long genes by FISH (doi:10.1038/s41556-022-00847-6; see Fig. 7). Another study showed that splicing inhibition decreased phosphorylation level of Ser2 in Pol II CTD (<https://doi.org/10.1093/nar/gkv740>).
It is imperative for authors to contextualize their findings within the framework of these prior studies. I strongly advocate conducting a Western blot analysis to investigate the impact of U2af38 and SRPK downregulation on Pol II CTD phosphorylation. Should this analysis yield affirmative results, it suggests a plausible mechanism for the observed transcriptional attenuation: splicing-dependent phosphorylation of Pol II CTD.

Thank you for these comments, which are extremely helpful in improving the clarity of our manuscript---especially we are thankful for bringing relevant earlier studies to our attention. The reason we proposed ‘transcript entangling’ is that the transcription attenuation was so specific to gigantic genes (that is, short genes did not exhibit transcription attenuation in splicing mutants), accordingly, the model has to explain why only long genes’ transcription, but not short genes’ transcription, was impacted. Our model of ‘entanglement’ is one such possibility that can differentially impact long vs. short genes, and also implied by cytology. Indeed, our model and observation are consistent with the observation by (Sousa-Luis et al., 2021). Additionally, it is notable that (Leidescher et al., 2022) specifically chose large, lampbrush genes (but did not examine potential attenuation of small genes upon splicing inhibition), and it is possible that their observation is specific to such large, lampblush genes. Our revised discussion integrates these notions.

This reviewer suggested to examine the state of pol II (phosphor-Ser2). This suggestion is motivated by the idea that this may suggest ‘transcription elongation defects’ in explaining our observation (attenuation of sequence reads toward 3’ side). However, considering that only long genes are affected (only less than 10 genes), while many many more short genes are NOT impacted, it is highly unlikely that western blotting will give us a definitive answer, even if the reviewer’s model (which is plausible) is correct. (additionally, western blotting using entire testis tissue will contain pol II from mitotically amplifying spermatogonia as well, further obscuring the interpretation).

Therefore, we opted not to conduct western blotting as requested. We hope that the editor and the reviewers will understand the rationale and agree with us not conducting western blotting. We have revised the discussion to clarify the reasoning why we propose ‘transcript entangling model’, emphasizing specific impact on long genes (and thus ‘general Ser2 phosphorylation state’ is unlikely to explain the observation).

Reviewer #2: This article describes a novel function for co-transcriptional splicing: keeping the pre-mRNA transcript length of gigantic genes short to ensure proper expression. The Drosophila Y chromosome provides an excellent model for studying intron gigantism – the Y chromosome encodes only 20 genes, 6 of which are giant genes with large satellite DNA rich-introns. These Y-linked gigantic genes are only expressed during spermatogenesis, take days(!) to transcribe, and act as fertility factors. The authors’ use of hybridization chain reaction (HCR) RNA FISH to detect short target sequences in spermatocytes and visualize distinct transcription products (i.e. exon-intron products for nascent mRNA vs exon-exon products for spliced transcripts) is ingenious. With this technique, the authors confirm their previous work that Y-linked gigantic genes are transcribed as a single transcript and that transcription proceeds in the 5’-3’ order. Additionally, the authors demonstrate that these genes are co-transcriptionally spliced, and that RNAi-mediated depletion of critical splicing factors U2af38 and SRPK results in downregulation of Y-linked gigantic genes and sterility. The authors propose that splicing defects may lead to attenuation of transcription by preventing mRNAs from becoming too long and getting entangled. While the question remains whether giant introns serve any functional purpose, this work hints at the possibility that giant introns may facilitate differential gene expression when combined with specific expression of splicing factors.

This manuscript is well-written, easy to read, and accompanied by beautiful images. I have only minor comments:

We thank this reviewer for their positive comments. We have addressed their comments as following.

1. Line 105: In the text the authors mention that “kl-3 is co-transcriptionally spliced (Fig. 1E). Spliced exon 1 – exon 2 junctions were observed in early SCs alongside nascent exon 1 – intron 1 junctions (Fig. 1E, magenta and green arrowheads).” However, the data in Figure 1E shows green probe (exon 1 – intron 1) before the appearance of magenta (exon 1 – exon 2). Are both exon 1 – intron 1 junctions and spliced exon 1 – exon 2 junctions always detected in the same cells? If not, the authors should place the magenta arrow immediately to the right of the green arrow to reflect the earliest detection of each probe more accurately. This would still be consistent with their conclusion that Y-linked gigantic genes are co-transcriptionally spliced.

This reviewer is correct about the timing. We have moved the magenta arrowhead and we adjusted the description in the main text.

2. Line 546, Figure S1B: “exon3 – intron4” should be “exon 3 – exon4” to accurately reflect the probe target locations shown in the kl-3 gene diagram.

Thank you for catching this. We corrected.

3. Figure S1B and C: It is very difficult to see the dark blue FISH probes in printed versions of the manuscript (it is only slightly clearer on a computer) – the authors should consider using another color (cyan or white?) to make visualizing the data easier.

Thank you for the suggestion. White is used for DAPI channel, and cyan cannot be differentiated when merged with white. We have adjusted the brightness of blue to make it easier to see.

4. Figure 4: the letter B is missing from the panel.

Thank you for catching this. We added the letter B.

5. Line 196: The authors mentioned 1429 altered splicing events in SRPK RNAi – only 1420 are denoted in Figure S5C.

Thank you for catching this. It was a typo in the main text. We have corrected it.

6. Could inhibition of splicing lead to the appearance of cryptic polyadenylation signals in unspliced giant introns and result in premature transcription termination? If so, could this partially explain the drop in read depth that follows a giant intron (but not a smaller intron which may not contain cryptic polyadenylation signals)? It would be helpful if the authors clarify this point (note: I am not requesting an experiment as it would be beyond the scope of this work; a simple discussion point would suffice here).

We fully agree with this reviewer about this possibility. We have added possible alternative interpretations in the discussion.

References cited:

Leidescher, S., J. Ribisel, S. Ullrich, Y. Feodorova, E. Hildebrand, A. Galitsyna, S. Bultmann, S. Link, K. Thanisch, C. Mulholland, J. Dekker, H. Leonhardt, L. Mirny, and I. Solovei. 2022. Spatial organization of transcribed eukaryotic genes. *Nat Cell Biol*. 24:327-339.

Sousa-Luis, R., G. Dujardin, I. Zukher, H. Kimura, C. Weldon, M. Carmo-Fonseca, N.J. Proudfoot, and T. Nojima. 2021. POINT technology illuminates the processing of polymerase-associated intact nascent transcripts. *Mol Cell*. 81:1935-1950 e1936.
